# Hospitalizations for unexplained illnesses among U.S. veterans of the Persian Gulf War.

**DOI:** 10.3201/eid0402.980208

**Published:** 1998

**Authors:** J D Knoke, G C Gray

**Affiliations:** Naval Health Research Center, San Diego, California 92186, USA. knoke@nhrc.navy.mil

## Abstract

Persian Gulf War veterans have reported a variety of symptoms, many of which have not led to conventional diagnoses. We ascertained all active-duty U.S. military personnel deployed to the Persian Gulf War (552,111) and all Gulf War era military personnel not deployed (1,479,751) and compared their postwar hospitalization records (until 1 April 1996) for one or more of 77 diagnoses under the International Classification of Diseases (ICD-9) system. The diagnoses were assembled by the Emerging Infections Program, Centers for Disease Control and Prevention, and are here termed "unexplained illnesses." Deployed veterans were found to have a slightly higher risk of hospitalization for unexplained illness than the nondeployed. Most of the excess hospitalizations for the deployed were due to the diagnosis "illness of unknown cause" (ICD-9 code 799.9), and most occurred in participants of the Comprehensive Clinical Evaluation Program who were admitted for evaluation only. When the effect of participation in this program was removed, the deployed had a slightly lower risk than the nondeployed. These findings suggest that active-duty Gulf War veterans did not have excess unexplained illnesses resulting in hospitalization in the 4.67-year period following deployment.


### Hospitalizations for Unexplained Illnesses among U.S. Veterans of the Persian Gulf War

**James D. Knoke and Gregory C. Gray**
Naval Health Research Center, San Diego, California, USA

Persian Gulf War veterans have reported a variety of symptoms, many of which have not led to conventional diagnoses. We ascertained all active-duty U.S. military personnel deployed to the Persian Gulf War (552,111) and all Gulf War era military personnel not deployed (1,479,751) and compared their postwar hospitalization records (until 1 April 1996) for one or more of 77 diagnoses under the International Classification of Diseases (ICD-9) system. The diagnoses were assembled by the Emerging Infections Program, Centers for Disease Control and Prevention, and are here termed "unexplained illnesses." Deployed veterans were found to have a slightly higher risk of hospitalization for unexplained illness than the nondeployed. Most of the excess hospitalizations for the deployed were due to the diagnosis "illness of unknown cause" (ICD-9 code 799.9), and most occurred in participants of the Comprehensive Clinical Evaluation Program who were admitted for evaluation only. When the effect of participation in this program was removed, the deployed had a slightly lower risk than the nondeployed. These findings suggest that active-duty Gulf War veterans did not have excess unexplained illnesses resulting in hospitalization in the 4.67-year period following deployment.

The Persian Gulf War was one of the briefest full-scale conflicts in U.S. history. For a 2-month period of fighting ending in March 1991, nearly 700,000 U.S. service members were deployed to the Persian Gulf region. Since returning from the war, many veterans have reported unexplained symptoms (1-5), prompting allegations of a new disease or diseases (2,3,5,6). Numerous expert panels and research projects have examined illness and death among Gulf War veterans (4,7,8). However, with the exception of self-reported symptoms (8-14), no consistent pattern of increased illness or death has been reported (10,14-18).

The U.S. Department of Defense (DoD) conducted an epidemiologic comparison of the postwar DoD hospitalizations of service members deployed to the Gulf War and service members of the same era not deployed (15). In the 2-year period after the war, no consistent increase in the overall risk for hospitalization (or specific risk for hospitalization for various broad diagnostic categories) was found for those deployed. This study relied upon diagnoses based on the International Classification of Diseases, 9th Revision, Clinical Modification (ICD-9) system (19). Medical providers may not consistently classify a new or poorly recognized syndrome, and consequently a true difference in hospitalization risk could be spread across numerous diagnostic categories and remain undetected.

The present study compares the postwar DoD hospitalizations for diagnoses consistent with an unexplained illness of Persian Gulf War veterans and their nondeployed peers.

### Analytic Approach

### Study Population

The study population consisted of active-duty service members (Army, Air Force, Navy, Marine Corps, and Coast Guard) who were either deployed to the Persian Gulf War for 1 or more days during the Gulf War deployment period (8 August 1990 through 31 July 1991) or were not deployed but were on active duty for at least part of the Gulf War deployment period. All deployed (n = 552,111) and nondeployed (n = 1,479,751) service members who remained on active duty at the end of the period were included in the study population.

Address for correspondence: James D. Knoke, Naval Health Research Center, P.O. Box 85122, San Diego, CA 92186, USA; fax: 619-553-7601; e-mail: knoke@nhrc.navy.mil.

The study was restricted to active-duty personnel because they are rarely hospitalized outside DoD facilities. Members of the U.S. Reserve and National Guard forces were not studied because only a fraction of their postdeployment hospitalizations were in DoD facilities.

### Study Outcome

The study outcome was determined by 77 ICD-9 diagnoses assembled by the Emerging Infections Program, Centers for Disease Control and Prevention, to monitor death certificates for unexplained deaths (20). These diagnoses span several of the 17 major categories delineated in ICD-9 and include selected diagnoses from diseases of the blood, nervous system, circulatory system, respiratory system, and digestive system; infectious and parasitic diseases; and symptoms, signs, and ill-defined conditions. These diagnoses primarily relate to nonspecific infections and other ill-defined conditions, and for convenience they are termed “unexplained illnesses” in this study.

All study population admissions to U.S. military hospitals worldwide (reported to the DoD computerized hospitalizations database by 1 October 1996), subsequent to the Gulf War deployment period and before 1 April 1996, were evaluated for unexplained illnesses among as many as eight ICD-9 diagnoses coded for each admission. For another analysis, only the first coded diagnosis (the principal diagnosis) was ascertained. The DoD ICD-9 coding guidelines define the principal diagnosis as “the condition established, after study, to be responsible for the admission.” Hospitalizations prior to the conclusion of the deployment period were not evaluated because access to care for the deployed differed markedly from that for the nondeployed during this time. Outpatient visits were not studied because they are not computerized centrally by DoD.

### Data

Demographic variables available for use as covariates included age, race/ethnicity, occupation, rank, salary, branch of service, length of service, marital status, and gender. Time-dependent variables were evaluated as of 31 July 1990. A previous study (15) found hospitalization for any reason in the 12 months preceding the Gulf War deployment period to be an important predictor of postwar hospitalization. This indicator variable may be a surrogate for baseline health status and was also used as a covariate. Demographic data, including deployment status, were obtained from the Defense Manpower Data Center, Seaside, California. Hospitalization information was obtained from the Data Processing Center, Fort Detrick, Frederick, Maryland. Comprehensive Clinical Evaluation Program data were obtained from the Deployment Surveillance Team, Falls Church, Virginia.

### Statistical Analysis

Frequencies of selected diagnoses and causes of death were calculated. The Cox proportional hazards survival analysis model (21) was used to obtain the risk ratio (RR) and 95% confidence interval (CI) of deployment status (deployed relative to nondeployed) for an event consisting of hospitalization with an unexplained illness, adjusting for the covariates. Follow-up time was computed from 1 August 1991 until hospitalization in any DoD hospital worldwide with at least one unexplained illness, separation from the service, or until 31 March 1996, whichever occurred first. All data management and statistical calculations were performed with the Statistical Analysis System (22).

### Findings

### Frequent Unexplained Illnesses

Our study population consisted of 25,495 first hospitalizations (with at least one unexplained illness among the eight possible diagnoses), 6,672 in the deployed and 18,823 in the nondeployed. For these hospitalizations, the 10 most frequent first unexplained illnesses among all eight possible diagnoses were tabulated (Table 1). Eight of these diagnoses occurred with similar proportional distributions between the two groups. The diagnosis “nonspecific abnormal findings in the amniotic fluid” accounted for a higher proportion of hospitalizations among the nondeployed than among the deployed. This is consistent with a higher proportion of women among the nondeployed (12.7%) than among the deployed (6.1%). The tenth most frequent diagnosis, “illness of unknown cause,” accounted for a considerably greater proportion of hospitalizations among the deployed (8.3%) than among the nondeployed (1.8%).

An unexplained illness was the principal diagnosis in 13,490 first hospitalizations, 3,525

Table 1. Frequencies of the 10 most common diagnoses for first hospitalizations with an unexplained illness, considering all eight coded diagnoses[a]

| ICD-9 Code | Description | Frequency Total | Frequency Deployed | Frequency Non-deployed | Percentage of all diagnoses Deployed | Percentage of all diagnoses Non-deployed |
|---|---|---|---|---|---|---|
| 008.8 | Unspecified intestinal infections | 2286 | 581 | 1705 | 8.7 | 9.1 |
| 047.9 | Unspecified viral meningitis | 946 | 236 | 710 | 3.5 | 3.8 |
| 079.9 | Unspecified viral infections | 3540 | 896 | 2644 | 13.4 | 14.1 |
| 465.9 | Acute URI[b], unspecified site | 1999 | 527 | 1472 | 7.9 | 7.8 |
| 486 | Pneumonia, organism unspecified | 2840 | 719 | 2121 | 10.8 | 11.3 |
| 780.6 | Pyrexia of unknown origin | 961 | 233 | 728 | 3.5 | 3.9 |
| 785.6 | Enlarged lymph nodes | 1587 | 423 | 1164 | 6.3 | 6.2 |
| 786.0 | Dyspnea | 2736 | 703 | 2033 | 10.5 | 10.8 |
| 792.3 | Nonspecific abnormal amniotic fluid | 1317 | 188 | 1129 | 2.8 | 6.0 |
| 799.9 | Illness of unknown cause | 892 | 555 | 337 | 8.3 | 1.8 |
| Subtotal | | 19,104 | 5061 | 14,043 | 75.9 | 74.6 |
| Other | | 6391 | 1611 | 4780 | 24.1 | 25.4 |
| Total | | 25,495 | 6672 | 18,823 | 100.0 | 100.0 |

[a]Only the first unexplained illness coded for each hospitalization was tabulated.
[b]URI, upper respiratory infection.

from the deployed and 9,965 from the nondeployed. The proportional distributions of the 10 diagnoses reported in Table 1 showed some rearrangements when only the principal diagnosis was considered (Table 2). The percentages of "acute upper respiratory infection, unspecified site"; "nonspecific abnormal amniotic fluid"; and "illness of unknown cause" were dramatically lower as the principal diagnosis than they were as any diagnosis. However, "illness of unknown cause" remained the only diagnosis that accounted for an appreciably larger proportion of admissions among the deployed (2.0%) than among the nondeployed (0.3%).

### Screening of Covariates

The demographic characteristics of the deployed and nondeployed groups have been reported (15). All available covariates were included in a preliminary Cox proportional hazards model to assess their effect on the risk for hospitalization for an unexplained illness (Table 3). All available covariates (one variable at a time) were also included in a series of preliminary models for this same purpose. These two approaches to screening covariates gave somewhat different results, probably because of multicolinearity among the covariates. The second approach was less useful than the first, so it is not further reported here.

Given the other covariates, age, marital status, and length of service were only minimally related to risk (possibly because of colinearity) and were not included in subsequent model analyses. The covariates retained included race (coded as white; black; or other, including unknown); rank (coded as enlisted or warrant or commissioned officer, including unknown); salary (coded as less than $1,000; $1,000 to $1,399; or at least $1,400 a month, including unknown); and branch of service (coded as Army or other). The military system has a large number of occupational categories; however, only "health-care worker" appeared to have an appreciably higher risk for hospitalization for an unexplained illness than other occupational categories. Consequently, occupation was simply coded as health-care worker or other (including unknown). Prewar hospitalization status was coded as "yes" if one was hospitalized for any reason during the 12 months before 1 August 1990 and as "no" otherwise. All of these other covariates, as well as gender (with unknowns included with men), had a highly statistically significant effect on the risk for hospitalization for an unexplained illness and were included in all subsequent model analyses. Other possible covariates, whose effect on risk was either less highly significant or nonsignificant, were not included so as to minimize unnecessary computation and variance inflation.

### Survival Analysis, Using All Eight Diagnoses

A model analysis including deployment status and the selected covariates showed that deployment status was significantly associated with hospitalization for an unexplained illness

Table 2. Frequencies of the 10 most common diagnoses for first hospitalization with an unexplained illness, considering only the principal diagnosis

| ICD-9 Code | Description | Frequency Total | Frequency Deployed | Frequency Non-deployed | Percentage of all diagnoses Deployed | Percentage of all diagnoses Non-deployed |
|---|---|---|---|---|---|---|
| 008.8 | Unspecified intestinal infections | 1807 | 467 | 1340 | 13.2 | 13.5 |
| 047.9 | Unspecified viral meningitis | 912 | 228 | 684 | 6.5 | 6.9 |
| 079.9 | Unspecified viral infections | 2620 | 661 | 1959 | 18.7 | 19.8 |
| 465.9 | Acute URI, unspecified site | 487 | 151 | 336 | 4.3 | 3.4 |
| 486 | Pneumonia, organism unspecified | 2350 | 615 | 1735 | 17.5 | 17.4 |
| 780.6 | Pyrexia of unknown origin | 382 | 93 | 289 | 2.6 | 2.9 |
| 785.6 | Enlarged lymph nodes | 1000 | 274 | 726 | 7.8 | 7.3 |
| 786.0 | Dyspnea | 1176 | 302 | 874 | 8.6 | 8.8 |
| 792.3 | Nonspecific abnormal amniotic fluid | 5 | 1 | 4 | 0.0 | 0.0 |
| 799.9 | Illness of unknown cause | 101 | 72 | 29 | 2.0 | 0.3 |
| Subtotal | | 10,840 | 2864 | 7976 | 81.2 | 80.0 |
| Other | | 2650 | 661 | 1989 | 18.8 | 20.0 |
| Total | | 13,490 | 3525 | 9965 | 100.0 | 100.0 |

when all eight possible diagnoses were used (Table 4). Separate model analyses for the two deployment status groups indicated that the parameter estimates were similar, and the effect of the covariates on the probability of hospitalization was essentially independent of deployment status.

The probabilities under the separate models of hospitalization with an unexplained illness, at the mean values of the included covariates, are presented as a function of follow-up time in Figure 1. These probabilities for the two groups were virtually coincidental and linear over time, until late 1994 when the deployed group's probability increased. This shift in the historical track for the deployed group suggests that the hazards for the two groups were not proportional over time and that additional analyses may be indicated.

### The Comprehensive Clinical Evaluation Program

In May 1994, DoD announced a Comprehensive Clinical Evaluation Program (CCEP), offering thorough clinical examinations and evaluations to Gulf War veterans who sought them. This program was implemented in June 1994. The divergence in probability curves (Figure 1) in the last quarter of 1994 prompted us to investigate whether the introduction of CCEP may have affected the probability of hospitalization.

Referring to any hospitalization for unexplained illness after 1 June 1994 of a CCEP participant as a CCEP hospitalization, we found that 837 first unexplained illness hospitalizations in the deployed were CCEP hospitalizations, as were 55 in the nondeployed. (For the purpose of CCEP participation, deployment status was self-determined.) Many of the nondeployed (according to Defense Manpower Data Center data) CCEP participants may have been in the Persian Gulf region after 1 August 1991. Of these 892 hospitalizations, 59% of the first diagnoses for unexplained illnesses were "illness of unknown cause," the most nonspecific diagnosis in ICD-9. Furthermore, with 128 DoD hospitals operating worldwide in 1995, 60% of these hospitalizations were in six facilities—three Army hospitals and three Air Force hospitals—and fewer than 1% were in any Navy facility.

These unusual results prompted us to make inquiries at some of the DoD hospitals reporting large numbers of hospitalizations for "illness of unknown cause." We learned that several of the larger Army and Air Force hospitals established special wards for CCEP participants reaching phase 2 of the evaluation process, admitted them for several days, and performed extensive evaluations (including invasive procedures and sleep studies) during hospitalization. These facilities also gave at least some of these participants a diagnosis of "illness of unknown cause." This coding practice for evaluation admissions may have resulted from a memo dated 25 August 1994 from the Headquarters,

Table 3. Frequencies and risk ratios under the Cox proportional hazards model for all hospitalizations with at least one unexplained illness, all covariates, 1 August 1991 to 1 April 1996

| Variable | | Deployed | Nondeployed | Risk ratio[a] | 95% Confidence interval |
|---|---|---|---|---|---|
| Not deployed | | | 1,479,751 | | |
| Deployed | | 552,111 | | 1.08 | 1.05 - 1.11 |
| Not hospitalized before war[b] | | 513,486 | 1,375,169 | | |
| Hospitalized before war | | 38,625 | 104,582 | 1.73 | 1.66 - 1.79 |
| Not married | | 258,821 | 602,553 | | |
| Married | | 291,009 | 875,309 | 1.05 | 1.02 - 1.08 |
| Unknown marital status | | 2282 | 1889 | 0.64 | 0.41 - 1.00 |
| Male | | 517,223 | 1,291,323 | | |
| Female | | 33,690 | 188,273 | 2.13 | 2.06 - 2.20 |
| Unknown gender | | 1198 | 155 | 0.60 | 0.20 - 1.75 |
| White | | 383,704 | 1,110,949 | | |
| Black | | 130,249 | 286,427 | 1.05 | 1.02 - 1.08 |
| Hispanic | | 12,900 | 26,696 | 0.91 | 0.83 - 1.00 |
| Other | | 23,592 | 54,128 | 0.91 | 0.85 - 0.97 |
| Unknown race/ethnicity | | 1666 | 1551 | 1.08 | 0.69 - 1.68 |
| Age in years: | 17-21 | 142,547 | 343,535 | | |
| | 22-25 | 146,652 | 306,272 | 0.94 | 0.90 - 0.97 |
| | 26-31 | 138,173 | 373,698 | 0.95 | 0.90 - 1.00 |
| | ≥ 32 | 123,353 | 455,302 | 1.05 | 0.99 - 1.12 |
| | Unknown | 1386 | 944 | 1.04 | 0.57 - 1.88 |
| Infantry, gun crews, seamanship | | 140,742 | 273,079 | | |
| Electronic equipment repair | | 45,263 | 159,195 | 0.99 | 0.93 - 1.04 |
| Communications/intelligence | | 55,665 | 120,108 | 1.00 | 0.95 - 1.06 |
| Health care | | 28,398 | 107,591 | 1.46 | 1.39 - 1.53 |
| Other technical | | 11,633 | 33,246 | 1.02 | 0.93 - 1.11 |
| Administration | | 62,194 | 255,576 | 0.96 | 0.92 - 1.00 |
| Electrical/mechanical repair | | 110,258 | 224,527 | 0.98 | 0.93 - 1.02 |
| Construction/related trades | | 19,424 | 47,800 | 1.11 | 1.03 - 1.20 |
| Supply handlers | | 53,966 | 113,093 | 1.11 | 1.06 - 1.17 |
| Trainees, undesignated | | 6421 | 64,625 | 1.02 | 0.95 - 1.10 |
| Unknown job category | | 18,147 | 80,911 | 1.18 | 1.11 - 1.25 |
| Enlisted | | 489,034 | 1,238,790 | | |
| Warrant Officer | | 7760 | 13,555 | 0.79 | 0.69 - 0.89 |
| Commissioned Officer | | 53,121 | 227,381 | 0.70 | 0.65 - 0.75 |
| Unknown status | | 2196 | 25 | 0.43 | 0.20 - 0.93 |
| Salary/month: | < $1000 | 122,161 | 328,187 | | |
| | $1000-$1399 | 269,500 | 573,995 | 0.81 | 0.77 - 0.85 |
| | $1400-$2099 | 75,523 | 252,923 | 0.68 | 0.63 - 0.72 |
| | $2100-$3199 | 60,528 | 200,295 | 0.73 | 0.68 - 0.79 |
| | ≥$3200 | 22,203 | 124,326 | 0.74 | 0.66 - 0.83 |
| | Unknown | 2196 | 25 | – | – – |
| Army | | 258,858 | 459,644 | | |
| Navy | | 141,604 | 423,138 | 0.61 | 0.59 - 0.64 |
| Marine Corps | | 83,377 | 113,467 | 0.66 | 0.63 - 0.70 |
| Air Force | | 67,942 | 447,125 | 0.76 | 0.73 - 0.78 |
| Coast Guard | | 330 | 36,377 | 0.42 | 0.37 - 0.48 |
| Service in months: | < 22 | 124,629 | 262,260 | | |
| | 22-54 | 162,123 | 269,727 | 1.05 | 1.00 - 1.11 |
| | 55-126 | 133,608 | 363,138 | 0.97 | 0.92 - 1.02 |
| | ≥ 127 | 131,751 | 584,626 | 1.12 | 1.07 - 1.18 |

[a]Risk ratios are relative to the first group in each variable category. When the algorithm failed to converge, because of lack of cases in the category, a dash (–) is given.

[b]In the 12 months preceding 1 August 1990.

Table 4. Frequencies and risk ratios under the Cox proportional hazards model for all hospitalizations with at least one unexplained illness, selected demographic variables, 1 August 1991 to 1 April 1996

| Variable | | Deployed | Nondeployed | Risk ratio[a] | 95% Confidence interval |
|---|---|---|---|---|---|
| Not deployed | | — | 1,479,751 | | |
| Deployed | | 552,111 | — | 1.06 | 1.03 - 1.09 |
| Not hospitalized before war[b,c] | | 513,486 | 1,375,169 | | |
| Hospitalized before war | | 38,625 | 104,582 | 1.71 | 1.65 - 1.78 |
| Male[c] | | 518,421 | 1,291,478 | | |
| Female | | 33,690 | 188,273 | 2.11 | 2.04 - 2.18 |
| White | | 383,704 | 1,110,949 | | |
| Black | | 130,249 | 286,427 | 1.04 | 1.01 - 1.08 |
| Other race/ethnicity[c] | | 38,158 | 82,375 | 0.91 | 0.86 - 0.96 |
| All other occupations[c] | | 523,713 | 1,372,160 | | |
| Health-care worker | | 28,398 | 107,591 | 1.44 | 1.38 - 1.50 |
| Enlisted | | 489,034 | 1,238,790 | | |
| Officer[c] | | 63,077 | 240,961 | 0.66 | 0.62 - 0.70 |
| Salary/month: | < $1,000 | 122,161 | 328,187 | | |
| | $1,000-$1,399 | 269,500 | 573,995 | 0.77 | 0.74 - 0.79 |
| | ≥ $1,400[c] | 160,450 | 577,569 | 0.82 | 0.78 - 0.87 |
| Army | | 258,858 | 459,644 | | |
| Other branches | | 293,253 | 1,202,107 | 0.60 | 0.58 - 0.62 |

[a]Risk ratios are relative to the first category for each variable.
[b]In the 12 months preceding 1 August 1990.
[c]Includes unknown.

U.S. Army Medical Command at Fort Sam Houston, San Antonio, Texas, which directed Army facilities to code CCEP participants with "unexplained complaints with no confirmed diagnosis" as 799.9. The practice of admitting CCEP participants was discontinued by mid-1995. The probability of hospitalization curves tended to become parallel again about mid-1995 (Figure 1). Thus, we inferred that a substantial majority of the CCEP hospitalizations were primarily for evaluation.

Independent evaluation supported this inference. Since DoD computerized hospital records do not include cause of hospitalization or any other indication of whether a patient may have been hospitalized primarily for evaluation, we randomly selected for chart review 50 CCEP hospitalizations from each of the Army and Air Force facilities with the greatest numbers of CCEP hospitalizations. Both of these facilities were located in the same metropolitan area, San Antonio, Texas. The selected CCEP hospitalizations were independently evaluated by two clinicians, one from each facility. Seventy-nine charts were reviewed, 44 from the Air Force facility and 35 from the Army facility. The other 21 records were located in satellite facilities or otherwise not readily accessible. The two clinicians agreed that 77 of these hospitalizations were for evaluation only and would not have been considered hospitalizations had the CCEP not been in effect. One of the clinicians thought that two of the hospitalizations were for clinical management and would have occurred regardless of CCEP.

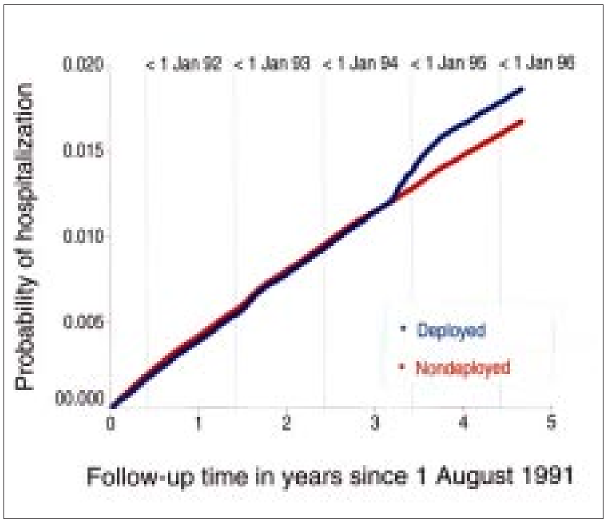

Figure 1. Probability of hospitalization for unexplained illness, deployed and nondeployed veterans.

### Survival Analysis, Using All Eight Diagnoses, Censoring CCEP Participants

We repeated the survival analyses (reported in Table 4; Figure 1), censoring all CCEP participants on 1 June 1994. This change resulted in 5,835 first hospitalizations for an unexplained illness in the deployed group and 18,768 in the nondeployed. No important differences were observed in the effects of the covariates between the models censoring CCEP participants on 1 June 1994 and those reported in Table 4. However, when CCEP participants were censored, the risk for hospitalization for an unexplained illness was lower in the deployed than in the nondeployed (RR = 0.93, CI = 0.91 to 0.96), and the probability of hospitalization for an unexplained illness was generally lower over time for the deployed than for the nondeployed (Figure 2). Also, the proportional hazards assumption now appeared reasonable.

CCEP participants may truly have been at increased risk for hospitalization for an unexplained illness, in spite of many CCEP hospitalizations having been for evaluation only. Consequently, the true risk for the deployed is likely to be intermediate to that depicted in Figures 1 and 2. However, our record review, which concluded that the preponderance of CCEP hospitalizations were for evaluation only, suggested that the results are more closely depicted in Figure 2 than Figure 1.

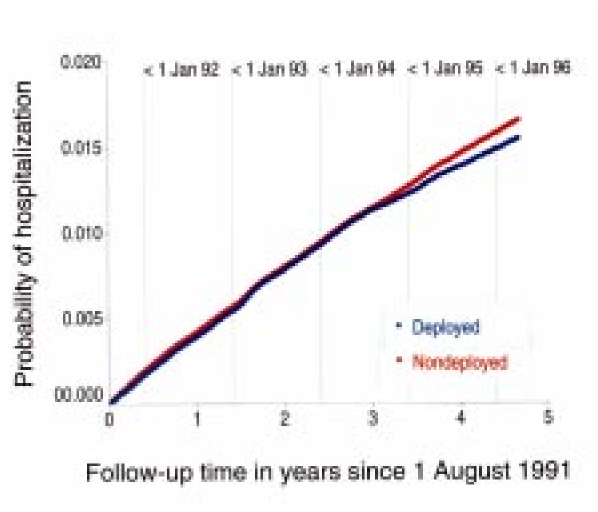

Figure 2. Probability of hospitalization for unexplained illness, deployed and nondeployed veterans, censoring comprehensive clinical evaluation program participants on 1 June 1994.

### Survival Analyses, Using Principal Diagnosis Only

Model analysis of hospitalizations for which the principal diagnosis was an unexplained illness showed that deployment status was not a statistically significant predictor (RR = 0.99, CI = 0.95 to 1.03). When CCEP participants were censored on 1 June 1994, model analysis results were similar and showed that the deployed were slightly less likely than the nondeployed to be hospitalized (RR = 0.93, CI = 0.89 to 0.97). Model analysis results for hospitalization for an unexplained illness using only the principal diagnosis thus were similar to the results when all eight diagnoses were employed, except that there were many fewer admissions with “illness of unknown cause” as a principal diagnosis than as a secondary diagnosis.

### Deaths

During hospitalization with an unexplained illness 348 veterans died. Of those who died, 86 had been deployed, and 262 had not. Of the 348 deaths, only 62 had an unexplained illness indicated as the underlying cause. Of these 62, 17 had been deployed and 45 had not. Deployed veterans with an unexplained illness as the underlying cause of death included four with “unspecified septicemia”; six with “pneumonia, organism unspecified”; three with “other diseases of the lung”; and one each with “other primary cardiomyopathies,” “thrombotic microangiopathy,” “unexplained death,” and “respiratory failure.”

### Conclusions

Because they live in close quarters and are required to travel, military personnel commonly acquire diseases endemic to the regions they visit and may serve as reservoirs and vectors for disease transmission when they return home. A number of military deployment-related epidemics have occurred in recent years. Persian Gulf War veterans have been diagnosed with a new manifestation of leishmaniasis (23). Veterans of the peace-keeping effort in Somalia have had increased hospitalization rates for malaria (24), deployed Navy personnel have brought nonendemic strains of HIV virus into the United States (25), and Russian soldiers have been implicated in the mass epidemics of diphtheria in Eastern Europe (26). These and other observations make surveillance for unexplained illnesses among U.S. military personnel an important federal public health issue.

We designed this study to screen for unexplained illnesses using a collection of ICD-9 diagnoses (20). Since unexplained illnesses generally have no specific ICD-9 diagnoses, a nonspecific diagnosis from this collection is likely to be used by hospital coders when an unexplained illness is encountered. The DoD worldwide hospitalization data subsequent to the Persian Gulf War were analyzed using the Cox proportional hazards model for evidence of unexplained illnesses. Analyses were performed both with all eight coded diagnoses and with only the principal diagnosis.

Model analysis showed that Gulf War veterans were more likely than their peers to be hospitalized for unexplained illness. After 4.67 years of follow-up, the cumulative probability of hospitalization for unexplained illness was 0.0185 in the deployed and 0.0166 in the nondeployed (Figure 1). This increased hospitalization risk of 11% for the deployed was a consequence of the recruiting for free clinical evaluations beginning in June 1994, with most of the resulting CCEP hospitalizations being for medical evaluation and not for clinical management. When CCEP participants were censored on 1 June 1994, deployed Gulf War veterans were not at greater risk than those not deployed. The slightly lower hospitalization risk for the deployed than for the nondeployed (Figure 2) is consistent with a healthy service member effect; that is, those selected for deployment are, on average, slightly healthier than those not selected.

This study had a number of limitations. Some miscoding of Gulf War deployment status was suggested by the finding that some nondeployed veterans were evaluated under the CCEP as being Gulf War veterans. However, veterans who did not serve in the Gulf region until after 31 July 1991 were eligible for the CCEP, and this apparent discrepancy was less than 6%. Also, many personnel separated from the service during the period of follow-up; 59.8% of the deployed and 54.3% of the nondeployed had separated by 1 April 1996. However, the fact that separating veterans receive thorough medical screening and have the potential of receiving lifelong disability benefits motivates them to be thorough in their reporting of illness before separation. The broad categorization of deployment status may have masked illness due to time- and geography-specific exposures, and illnesses not serious enough to require hospitalization have not been captured by our analyses. Finally, the collection of diagnoses used as the outcome measure was not designed specifically for our purposes, but it does have the advantage of prior development by other researchers (20).

This study also had its strengths. The large group sizes, along with the availability of numerous important covariates (demographic variables and prewar hospitalization), offer unusually high statistical power for the detection of differences in the hospitalization risk between groups. Additionally, discharge diagnoses are thoroughly recorded and edited by DoD hospitals, and active-duty personnel have few opportunities to be hospitalized outside the DoD system, which ensures high-quality data with few missing values.

In summary, these analyses show a slightly greater hospitalization risk for unexplained illness among deployed Gulf War veterans than among those not deployed. However, the excess hospitalizations for the deployed are attributable to participation in CCEP; most of these hospitalizations were for medical evaluation, not clinical management. Consequently, before initiation of CCEP, active-duty Gulf War veterans were not at increased risk for hospitalization for unexplained illness.

### Acknowledgments

We thank Drs. Edwin C. Matthews and Gregg T. Anders for completing the record review of the 79 randomly selected CCEP hospitalizations. We also thank Michael A. Dove and Wayne F. Woo for their assistance in obtaining the deployment status and covariate data.

This is Naval Health Research Center report no. 96-35, supported by the Department of Defense (Health Affairs) and the Naval Medical Research and Development Command, Department of the Navy, Bethesda, Maryland, under work unit no. 63738DP4464.001-6423.

James D. Knoke is statistician, Emerging Illness Division, Health Sciences and Epidemiology Department, Naval Health Research Center, San Diego. His work concentrates on methodology and applications in clinical trials and epidemiology. He has special interests in the areas of survival analysis, longitudinal analysis, multivariate analysis, and nonparametric methods. He serves as an investigator in a number of epidemiologic studies among Persian Gulf War veterans.

Captain Gregory C. Gray is head, Emerging Illness Division, Health Sciences and Epidemiology Department, Naval Health Research Center, San Diego. He is a medical epidemiologist, specializing in general preventive medicine and public health. He has experience conducting epidemiologic investigations among military populations, particularly those involving respiratory pathogens. He serves as principal investigator for a number of large epidemiologic studies among Persian Gulf War veterans.

### References

1. Cotton P. Gulf War symptoms remain puzzling. JAMA 1992;268:2619.
2. Why are we sick? Army Times 1994 Apr 25:12-23.
3. Cowley G, Hager M, Liu M. Tracking the second storm. Newsweek 1994 May 16:56-7.
4. Persian Gulf Veterans Coordinating Board. Unexplained illnesses among Desert Storm veterans: a search for causes, treatment, and cooperation. Arch Intern Med 1995;155:262-8.
5. Brown D. Diagnosis unknown: Gulf War syndrome. The Washington Post 1994 Jul 24;Sect. A:1 (col. 2-4).
6. Nicolson GL, Rosenberg-Nicolson NL. Doxycycline treatment and Desert Storm. JAMA 1995;273:618-9.
7. Research Working Group of the Persian Gulf Veterans Coordinating Board. Federally Sponsored Research on Persian Gulf Veterans' Illnesses for 1995. Washington: Department of Veterans Affairs; 1996. Annual Report to Congress.
8. Institute of Medicine. Health consequences of service during the Persian Gulf War: recommendations for research and information systems. Washington: National Academy Press; 1996.
9. Unexplained illness among Persian Gulf veterans in an Air National Guard unit: Preliminary report—August 1990-March 1995. MMWR Morb Mortal Wkly Rep 1995;44:443-7.
10. DeFraites RF, Wanat ER, Norwood AE, Williams S, Cowan D, Callahan D. Investigation of a suspected outbreak of an unknown disease among veterans of Operation Desert Shield/Storm, 123rd Army Reserve Command, Fort Benjamin Harrison, Indiana, April 1992. Washington: Epidemiology Consultant Service, Division of Preventive Medicine, Walter Reed Army Institute of Research; 1992.
11. Presidential Advisory Committee on Gulf War Veterans' Illnesses. Final Report, Dec 1996. Washington: U.S. Government Printing Office; 1996.
12. Defense Science Board. Report of the Defense Science Board Task Force on Persian Gulf War Health Effects. Washington: Office of the Under Secretary of Defense for Acquisition and Technology; 1994.
13. National Institutes of Health Technology Assessment Workshop Panel. The Persian Gulf experience and health. JAMA 1994;272:391-6.
14. Kaiser KS, Hawksworth AW, Gray GC. A comparison of self-reported symptoms among active-duty Seabees: Gulf War veterans versus era controls. In: Proceedings of the 123rd Annual Meeting of the American Public Health Association; 1995 Oct 31; San Diego, California. Washington: American Public Health Association; 1995.
15. Gray GC, Coate BD, Anderson CM, Kang HK, Berg SW, Wignall FS, et al. The postwar hospitalization experience of U.S. Persian Gulf War veterans compared with other veterans of the same era. N Engl J Med 1996;335:1505-13.
16. Kang HK, Bullman TA. Mortality among U.S. veterans of the Persian Gulf War. N Engl J Med 1996;335:1498-504.
17. Writer JV, DeFraites RF, Brundage JF. Comparative mortality among U.S. military personnel in the Persian Gulf region and worldwide during Operations Desert Shield and Desert Storm. JAMA 1996;275:118-21.
18. Cowan DN, DeFraites RF, Wishik SM, Goldenbaum MB, Wishik SM. The risk of birth defects among children of Gulf War veterans. N Engl J Med 1997;336:1650-6.
19. The International Classification of Diseases, 9th Revision, Clinical Modification. 3rd ed. Washington: U.S. Department of Health and Human Services; 1991.
20. Perkins BA, Flood JM, Danila R, Holman RC, Reingold AL, Klug LA, et al. Unexplained deaths due to possibly infectious causes in the United States: defining the problem and designing surveillance and laboratory approaches. Emerg Infect Dis 1996;2:47-53.
21. Kalbfleisch JD, Prentice RL. The Statistical Analysis of Failure Time Data. New York: John Wiley & Sons; 1980.
22. SAS Institute Inc. SAS Language Reference. Version 6. 1st ed. Cary (NC): SAS Institute Inc.; 1990.
23. Magill AJ, Grogl M, Gasser RA Jr, Sun W, Oster CN. Visceral infection caused by *Leishmania tropica* in veterans of Operation Desert Storm. N Engl J Med 1993;328:1383-7.
24. Wallace MR, Sharp TW, Romajzl PJ, Batchelor RA, Thornton SA, Longer CF, et al. Malaria in Mogadishu, Somalia. Clin Infect Dis 1993;17:510-1.
25. Brodine SK, Mascola JR, Weiss PJ, Ito SI, Porter KR, Artenstein AW, et al. Detection of diverse HIV-1 genetic subtypes in the USA. Lancet 1995;346:1198-9.
26. Maurice J. Russian chaos breeds diphtheria outbreak. Science 1995;267:1416-7.
